# Editorial: Small molecules and peptides in paracrine/autocrine regulation of ovarian folliculogenesis

**DOI:** 10.3389/fendo.2023.1168701

**Published:** 2023-03-03

**Authors:** So-Youn Kim, Jing Xu, Artur Mayerhofer, Cecily V. Bishop

**Affiliations:** ^1^ Olson Center for Women’s Health, Department of Obstetrics and Gynecology, College of Medicine, University of Nebraska Medical Center, Omaha, NE, United States; ^2^ Department of Biology & Chemistry, School of Health Sciences, Liberty University, Lynchburg, VA, United States; ^3^ BMC Munich, Cell Biology, Anatomy III, Ludwig-Maximilians-University, Planegg, Germany; ^4^ Department of Animal and Rangeland Sciences, College of Agricultural Sciences, Oregon State University, Corvallis, OR, United States

**Keywords:** ovary, folliculogenesis, small molecules, peptides, fertility

Ovarian folliculogenesis is a well-regulated process critical for developing competent oocytes for fertilization. It is also crucial for producing a hormonal milieu required to prepare the reproductive tract to support the developing embryo. Disruptions in folliculogenesis are associated with several disease states along with infertility; however, many details of the autocrine/paracrine factors controlling folliculogenesis remain unclear. This includes cell-specific production patterns, direct actions, physiological roles, and downstream signaling pathways within ovarian follicles. Manuscripts featured in the Frontiers Research Topic “*Small Molecules and Peptides in Paracrine/Autocrine Regulation of Ovarian Folliculogenesis*” attempt to clarify several of these processes.

The manuscript entitled “*Homozygous mutation of gsdf causes infertility in female Nile Tilapia*” byJiang et al. suggested that gonadal somatic cell-derived factor (Gsdf) as a member of the TGF-β superfamily is critical for fertility in fish. Based on the previous report that homozygous *gsdf* mutations in Japanese medaka and zebrafish resulted in infertile females (1), the authors showed that *gsdf* homozygous mutations using CRISPR/Cas9 caused infertility in Nile tilapia. The number of phase IV oocytes was lower in *gsdf* ^-/-^ female fish than that of wild-type fish. In addition, they observed that the mutated ovaries were hyperplastic with more phase I oocytes. The ovaries of XX *gsdf* mutation fish exhibited altered gene expression with a total of 395 differentially-expressed genes. The KEGG enrichment analysis showed that the altered 244 signaling pathways were mainly related to ovarian steroidogenesis. Transcriptome analysis identified altered expression of TGF-β signaling in mutant fishes. Genes such as *amh* (anti-Müllerian hormone) and *amhr2* (amh receptor 2) were upregulated, while *inhbb* (inhibin B) and *acvr2* (activin receptor 2) were downregulated. However, *cyp19a1a* (cytochrome P450, family 19, subfamily A, polypeptide 1a; aromatase) and serum estradiol concentrations were comparable. They found that Gsdf interacted with TGF-β type II receptors (Amhr2 and Bmpr2a/bone morphogenetic protein receptor 2a). Altogether, they showed that Gsdf functions with TGF-β signaling pathway to control follicular development and function in fish, suggesting that a small peptide in the ovary is critical for fertility.

Adrenomedullin 2/intermedin (ADM2/IMD) is a peptide family member of ADM, calcitonin gene-related peptides (a- and b-CGRPs), calcitonin, and amylin. It transduces signals through calcitonin receptor-like receptor and one of the three receptor activity-modifying proteins (RAMP1, 2, and 3). ADM2 is known for the regulation of vascular lumen enlargement in mice. In the ovary, it facilitates cell-cell interactions in cumulus-oocyte complexes (COCs) by improving the expression of cell cycle progression genes like cyclin D2 (2). Furthermore, it has been known as a secretory factor controlling COC formation (3) and improving oocyte competence and embryo quality (4). The manuscript of Chang et al. expanded on these previous studies by reporting that disruption of intrafollicular ADM2 signaling led to follicular dysfunction using oocyte-specific conditional knockout mice with a *Zp3-Cre* promotor. The deletion of *Adm2* in oocytes increased the number of ovulated oocytes following a superovulation cycle, impaired the development capacity of fertilized eggs, and further decreased the size of the corpus luteum. They demonstrated that ADM2 is important to regulate hormonal response and follicle growth. Thus, this study further suggests that this peptide molecule contributes highly to normal ovarian function.

Two review articles are featured on this topic. The first by Luo et al. focuses on the roles of nitric oxide (NO) and reproductive biology. This article provides information on the known roles of NO in many aspects of male and female reproduction, mainly through the modulation of intracellular cGMP levels. In cells, NO is generated by three known forms of nitric oxide synthase (NOS): neuronal NOS (nNOS), endothelial NOS (eNOS), and inducible NOS (iNOS). While mostly associated with inflammation and immune responses, iNOS is expressed in granulosa cells and given the inflammatory nature of ovulation, this is a logical area of investigation. But all three forms of NOS are detected in granulosa cells of several species. The authors detailed the known actions of NO in follicles during the preantral stage and ovulation as well as the impacts of altered intracellular NO production/signaling during follicular atresia/apoptosis. These studies demonstrate that small molecule alterations to the follicular microenvironment deserve greater attention for their contributions to infertility and associated disease states.

The second review article by Zheng et al. details the available literature on another peptide known for regulating ovarian folliculogenesis. Growth differentiation factor 8 (GDF-8) is a member of the TGF-β superfamily and highly expressed in the ovary as well as skeletal muscle, adipose tissue, and cardiomyocytes. It regulates ovarian reproductive activities such as folliculogenesis, ovulation, and early embryo implantation. GDF-8 is expressed specifically in oocytes of primordial follicles and granulosa cells of antral follicles, as well as large and small luteal cells of the corpus luteum. It plays an important role in the production of estrogen in the ovary (5). Furthermore, GDF-8 regulates gonadotropin responsiveness by upregulating follicle stimulating hormone receptor (*FSHR*) expression (6). Meanwhile, GDF-8 is known as an inhibitory molecule in the regulation of progesterone production in the human ovary *via* downregulating steroidogenic acute regulatory protein (*STAR*) expression, which is critical for transporting cholesterol from the outer to the inner mitochondrial membrane (6). GDF-8 modulates the cumulus oophorus expansion by downregulating pentraxin 3 (*PTX3*) expression (7). Furthermore, GDF-8 impairs glucose metabolism, compromising granulosa cell proliferation and oocyte development. Most of all, the blood level of GDF-8 dynamically changes when patients undergo controlled ovarian hyperstimulation (7), suggesting the GDF-8 signaling pathway as a potential therapeutic for ovarian hyperstimulation syndrome (OHSS) and ovulation disorders like polycystic ovary syndrome (PCOS) (5, 8). Indeed, aberrant expression of GDF-8 is related to PCOS and OHSS. Thus, a regulatory peptide derived from the ovary contributes to ovarian and reproductive health.

The findings of these and other studies into the contribution of small molecules and peptides provide valuable insights into normal processes controlling folliculogenesis. These could also promote the identification of novel targets for drug discovery for contraceptives as well as for treatment of disorders associated with aberrant folliculogenesis including PCOS, OHSS, and ovarian aging.

## Author contributions

S-YK: Writing and editing manuscript JX: Writing and editing manuscript AM: Writing and editing manuscript CB: Writing and editing manuscript. All authors contributed to the article and approved the submitted version.
